# Characteristics of academic publications, preprints, and registered clinical trials on the COVID-19 pandemic

**DOI:** 10.1371/journal.pone.0240123

**Published:** 2020-10-06

**Authors:** Silvia Gianola, Tiago S. Jesus, Silvia Bargeri, Greta Castellini

**Affiliations:** 1 Unit of Clinical Epidemiology, IRCCS Istituto Ortopedico Galeazzi, Milan, Italy; 2 Global Health and Tropical Medicine (GHTM), WHO Collaborating Centre for Health Workforce Policy and Planning, Institute of Hygiene and Tropical Medicine—NOVA University of Lisbon, Lisbon, Portugal; Universitat Witten/Herdecke, GERMANY

## Abstract

The COVID-19 pandemic has unleashed a deluge of publications. For this cross-sectional study we compared the amount and reporting characteristics of COVID-19-related academic articles and preprints and the number of ongoing clinical trials and systematic reviews. To do this, we searched the PubMed database of citations and abstracts for published life science journals by using appropriate combinations of medical subject headings (MeSH terms), and the COVID-19 section of the MedRxiv and BioRxiv archives up to 20 May 2020 (21 weeks). In addition, we searched Clinicaltrial.gov, Chinese Clinical Trial Registry, EU Clinical Trials Register, and 15 other trial registers, as well as PROSPERO, the international prospective register of systematic reviews. The characteristics of each publication were extracted. Regression analyses and Z tests were used to detect publication trends and their relative proportions. A total of 3635 academic publications and 3805 preprints were retrieved. Only 8.6% (n = 329) of the preprints were already published in indexed journals. The number of academic and preprint publications increased significantly over time (p<0.001). Case reports (6% academic vs 0.9% preprints; *p*<0.001) and letters (17.4% academic vs 0.5% preprints; *p*<0.001) accounted for a greater share of academic compared to preprint publications. Differently, randomized controlled trials (0.22% vs 0.63%; *p*<0.001) and systematic reviews (0.08% vs 5%) made up a greater share of the preprints. The relative proportion of clinical studies registered at Clinicaltrials.gov, Chinese Clinical Trial Registry, and EU Clinical Trials Register was 57.9%, 49.5%, and 98.9%, respectively, most of which were still “recruiting”. PROSPERO listed 962 systematic review protocols. Preprints were slightly more prevalent than academic articles but both were increasing in number. The void left by the lack of primary studies was filled by an outpour of immediate opinions (i.e., letters to the editor) published in PubMed-indexed journals. Summarizing, preprints have gained traction as a publishing response to the demand for prompt access to empirical, albeit not peer-reviewed, findings during the present pandemic.

## Introduction

The pandemic spread of a novel human coronavirus, termed severe acute respiratory syndrome coronavirus 2 (SARS-CoV-2) by the International Committee on Taxonomy of Viruses [1] since late 2019 [2], has created a global health emergency. The urgent need to keep the public health response to COVID-19 informed by near-real-time monitoring and assessment [3] has brought with it the expansion of scientific effort and the production of new knowledge at an unprecedented rate [4]. There has been a parallel surge in the research being conducted, posted, and shared in the print and the digital media [5, 6]. The research progress in any area can be quantitatively estimated by bibliometric and scientometric analysis of publications that provides a comprehensive overview of trends in scientific research. Such analyses have been widely used for mapping scientific knowledge in diverse disciplines [7, 8]. For example, to better organize emerging and rapidly scientific developments, scoping reviews coupled with bibliometric and scientometric analysis have documented the networks of contributing authors, institutions, and countries [4, 6, 9, 10]. However, no reviews to date have focused on the epidemiology and the reporting characteristics of publications related to the COVID-19 pandemic [11, 12] or the advances in research during the pandemic [13–15].

Scientific information is distributed through various print and digital channels, among which are the formal publication sources such as registers of primary studies (i.e., trials) and systematic reviews, preprints, and academic publications. A clinical trials registry is a platform in which clinical trials are registered and cataloged. Clinical trials play an important role in response to the COVID-19 pandemic as a means to translate evidence from basic research into clinical practice [16, 17]. A systematic review protocol registry is an international database in which prospective systematic reviews in health and social care are listed. Systematic reviews should be registered at inception (at the protocol stage) to help avoid outcome reporting biases, publication bias, unplanned duplication and waste of resources, especially during the present global health emergency [18, 19]. Preprints are preliminary reports of work and have not yet undergone peer review. As such, they should not be relied on to guide clinical practice or health-related behavior and should not be reported in the news media as established information [20]. Preprint servers are open access online repositories that house preprint research articles by authors who choose to make their research immediately and freely available to the public. Research articles can receive commentary and peer review prior to journal submission, thus accelerating the dissemination of scientific findings [21, 22]. Preprints posted during the Ebola and Zika outbreaks divulged novel analyses and new data, while many of those that later appeared in academic publications were available for more than 100 days before publication [21]. Many preprints never undergo peer review; for instance, less than 5% of journal articles about the Ebola and the Zika virus were posted as preprints prior to publication in journals [21]. The information in preprints lacks the scrutiny and validity of an external, scientific review [23]. Nevertheless, analysis of preprints and ongoing investigations (trials and reviews) is essential for scientific advancement where timeliness is key, though peer reviews are still expedited for new relevant research.

With this context in mind, we wanted to take a snapshot of the amount of scientific development devoted to COVID-19 and gain an idea of the current status of global research. We also thought it necessary to obtain an insight into the reporting of future research, its amount, design, publication venues, and characteristics. To do this, we:

1. Compared the amount of COVID-19 related research in academic publications and preprints (not peer reviewed) stratified by key characteristics: species (*Humans*, *Animals*), study design (e.g., systematic review, randomized controlled trials—RCTs), and research area (e.g., drug treatment)
2. Evaluated the amount and key characteristics (e.g., research area) of ongoing COVID-19 research contained in clinical trials registers and systematic reviews.

## Methods

For this cross-sectional study, COVID-19 related-publications listed in electronic databases as of 20 May 2020 were reviewed.

### Data sources

We searched the PubMed database for COVID-19-related academic publications through its indexation system. PubMed is a comprehensive database that comprises more than 30 million citations (as of May 2020) for biomedical literature from MEDLINE, life science journals, and online books [24]. There is evidence in the health field that adding databases other than PubMed has only modest impact on the results of systematic reviews [25]. The medical subject headings (MeSH terms) in PubMed are organized in a hierarchical tree and assigned to each paper by subject-specialist indexers. For searching COVID-19-related articles, we used the supplementary concept records (SCRs). MEDLINE indexers regularly come across substances in the literature that are not currently MeSH headings. When this happens, the National Library of Medicine staff adds these substances to the MeSH vocabulary as SCRs. While MeSH headings are updated annually, new SCRs are added weekly. Therefore, COVID-19 articles are systemically indexed by research topic regardless of the specific words used by the authors. We conducted target searches in PubMed that included the use of appropriate combinations of MeSH terms and SCRs related to COVID-19 and related terms (e.g., SARS-CoV-2, coronavirus disease), filtered by species, research design, and research area (**S1 Appendix. PubMed database**).

We searched two popular sources for COVID-19-related preprints [26], the MedRxiv and BioRxiv databases, here defined as proxy indicators of published empirical not yet peer-reviewed literature. Articles on these preprints servers are examined by in-house staff who check for such issues as plagiarism and incompleteness [27] (**S1 Appendix. Preprints MedRxiv and BioRxiv databases**).

We then investigated all primary registries that meet the International Committee of Medical Journal Editors (ICMJE) criteria according to the WHO Registry Network. Primary registries in the WHO Registry Network meet specific criteria for content, quality, and validity, accessibility, unique identification, technical capacity and administration [28]. Additionally, we searched the PROSPERO international prospective register of systematic reviews database (**S1 Appendix. Primary register and PROSPERO register**).

All searches were run on 20 May 2020 (covering a 21-week period), without language restrictions.

### Data extraction

Data records retrieved from PubMed, MedRxiv, BioRxiv, trials registers, and PROSPERO databases were imported to Excel for further grouping and analysis. Data extraction was performed by one author and then checked by the second author. Any uncertainties were discussed between the data extractors.

For academic publications and preprints, we extracted the bibliometric data and then analyzed and summarized the subsets of the study or reporting characteristics: species (*Humans*, *Animals*); publication type (any type of article, systematic review, RCTs, epidemiologic studies, Letter to the Editor with or without original data); the distribution by research area (e.g., vaccine, drug treatment, rehabilitation, diagnostic testing, measures for infection prevention and control including social distance, masks); and biology according to the indexation facilities and the respective search tags or filters. Since the preprint servers have no such filters/tags, we manually extracted and coded the topics. Study designs such as laboratory experiments (e.g., genomics) and prognostic models were included in the epidemiology studies.

For the registers listing clinical trials, the following characteristics were filtered/tagged and extracted: country, study type, phase, recruiting status. Species and research area/tag were extracted from the records retrieved from the PROSPERO database.

### Data analysis

Descriptive statistics were initially used for analysis. The total number of publications for each data element (e.g., publication type), percentages, and frequencies were computed and displayed in tabular format, paired by academic and preprint sources. The total number of publications over time (weekly) was plotted onto a run chart. Simple linear regression with analysis of variance (ANOVA) was applied to analyze the growth in the number of COVID-19-related publications over time. Finally, two tailed two-sample Z-tests were performed to determine whether the proportion of each characteristic of the academic journal articles (indexed in PubMed) differed significantly from those contained in the preprint sources. Statistical significance was set at <0.05. Data analysis was performed using Stata Statistical Software. Release 16. (StataCorp LP, College Station, TX, USA).

## Results

### 1. Amount and characteristics of COVID-19-related research reported in academic and preprint (not peer reviewed) publications

**Fig 1:**
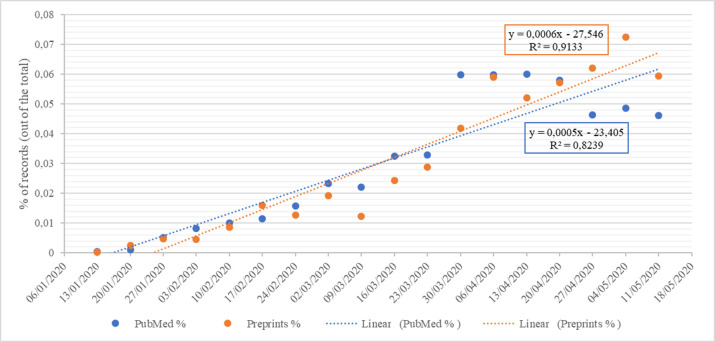
Linear regression over time for academic and preprint publications. Dates: 10 January 2020—first genomic data of its etiological agent (SARS-CoV-2); 20 January 2020—reports of confirmed cases from three countries outside China; 24 January 2020—first European case; 30 January 2020 –the WHO declares the novel coronavirus outbreak (2019-nCoV) a Public Health Emergency of International Concern (PHEIC); 11 March 2020 –the WHO declares a status of pandemic; 25 March 2020 –cases confirmed in all European Union/European Economic Area (EU/EEA) countries and in more than 150 countries worldwide.

**Fig 2:**
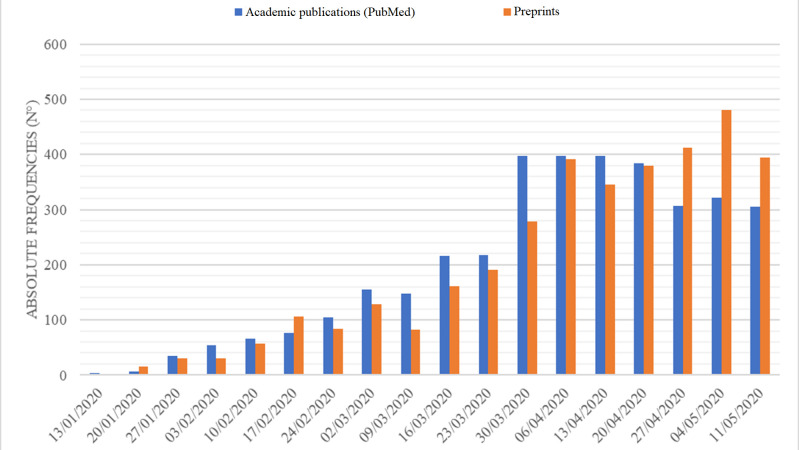
Absolute frequency of academic and preprint publications by week.

**Fig 3:**
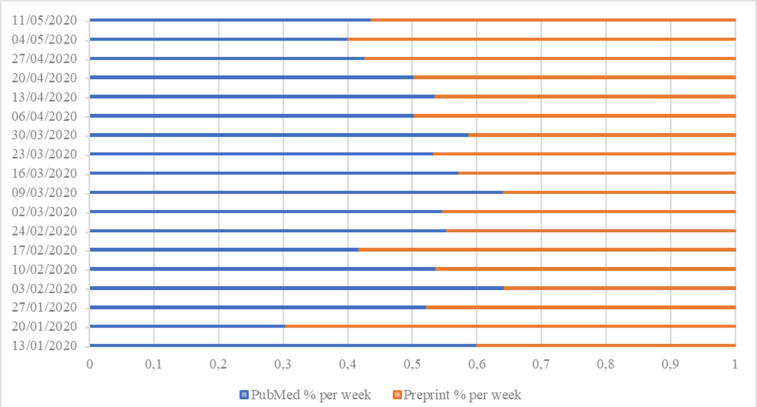
Relative distribution of academic and preprint publications by week.

**Fig 4:**
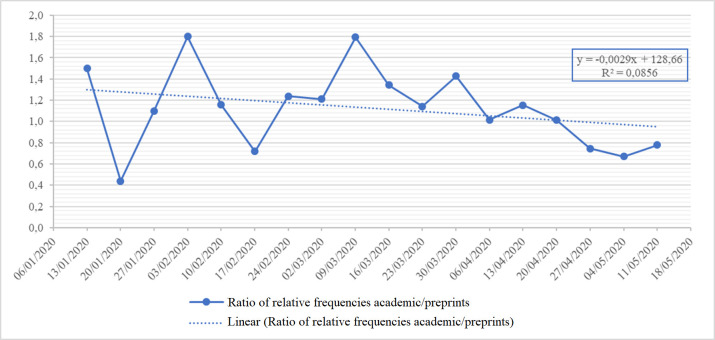
Linear regression of ratios between relative frequency (academic/preprint publications) by week.

From December 2019 through May 2020, a total of 7440 COVID-19-related publications were retrieved: 3635 (48.9%) academic publications and 3805 (51.1%) preprints. **Fig 1** presents the number of academic and preprint publications by week from December 2019 through May 2020. The increase in the number of academic and preprint publications was statistically significant for both types (academic *p<*0.001, r^2^ = 0.8239; preprints *p*<0.001, r^2^ = 0.9133). **Figs 2 and 3** present the absolute and the relative frequency of publications by week. **Fig 4** presents the trend of the ratio between the relative frequency of academic and preprint publications by week. There was no statistically significant difference in the rate of weekly increase in relative frequency between the two groups (*p* = 0.2388, r^2^ = 0.0856).

**Table 1** presents the reporting characteristics of academic and preprint COVID-19-related publications. In both types of publications, human subjects were predominant; the most mentioned research areas were prevention and control (26.1%, 950/3635 academic vs 42.4%, 1615/3805 preprints; *p*<0.001) and diagnosis (21.5%, 781/3635 academic vs 25.3%, 962/3805 preprints; *p*<0.001).

**Table 1:** Characteristics of COVID-19-related academic (PubMed) and preprint publications.

Characteristic		PubMed N = 3635 No. (%)	Preprints N = 3805 No. (%)	Z value	% Difference	P value
**Species**	*Humans*	3507 (96.5)	3634 (95.5)	2.14	1.0	<0.05
	*Animals*	128 (3.5)	93 (2.4)	2.74	1.1	<0.05
Publication type	Systematic review and/or meta-analysis	29 (0.8)	193 (5.0)	-10.83	-4.2	< 0.0001
	Randomized controlled trial	8 (0.2)	24 (0.6)	-2.71	-0.4	<0.05
	*Phase I*	1	0	1.02	1.0	>0.05
	*Phase II*	3	3	0.06	0.0	>0.05
	*Phase III*	0	2	-1.38	-2.0	>0.05
	*Phase IV*	0	3	-1.69	-3.0	>0.05
	*Protocols*	0	4	-1.96	-4.0	>0.05
	Epidemiologic studies	222 (6.1)	3522 (92.6)	-74.55	-86.5	< 0.0001
	*Observational studies*	21 (0.6)	940 (24.7)	-31.02	-24.1	< 0.0001
	Case report	219 (6.0)	35 (0.9)	12.12	5.1	< 0.0001
	Letter to the Editor	632 (17.4)	19 (0.5)	25.77	16.9	< 0.0001
Research Area/Tag	Vaccine	101 (2.8)	114 (2.9)	-0.56	-0.1	>0.05
	Drug therapy	57 (1.6)	325 (8.5)	-13.62	-6.9	< 0.0001
	Diagnosis	781 (21.5)	962 (25.3)	-3.87	-3.8	< 0.0001
	Prevention and control (e.g., masks, social distancing)	950 (26.1)	1615 (42.4)	-14.80	-16.3	< 0.0001
	Rehabilitation (e.g., pulmonary rehabilitation)	23 (0.6)	5 (0.1)	3.53	0.5	< 0.0001
	Prognosis	123 (3.4)	844 (22.2)	-24.10	-18.8	< 0.0001
	Biology/Genetic	43 (1.1)	866 (22.8)	-28.41	-21.7	< 0.0001

*Note*: the sum of the characteristics does not correspond to the total number of publications since specifications or other may overlap.

There were statistically significant differences in the relative frequency of study types between academic and preprint publications. For instance, RCTs were significantly more frequently reported in preprints than in academic publications (academic 0.2%, 8/3635 vs. preprints 0.6%, 24/3805; *p*<0.001). Similarly, systematic reviews appeared significantly more often in preprints (academic 0.8%, 29/3635 vs. preprints 5.0%, 193/3805; *p*<0.001). Observational studies were significantly more common in preprint than in academic publications (academic 0.6%, 21/3635 vs. preprints 24.7%, 940/3805; *p*<0.001). In contrast, case reports and letters to the editor were significantly more common in academic publications (case reports in academic publications 6.0%, 219/3635 vs. preprints 0.9%, 35/3805; *p*<0.001; letters to the editor in academic publications 17.4%, 632/3635 vs. preprints 0.5%, 19/3805; *p*<0.001). Overall, in both types of publications the most often mentioned research areas were prevention and control (26.1%, 950/3635 vs. 42.4%, 1615/3805; *p*<0.001) and diagnosis (21.5%, 781/3635 vs. 25.3%, 962/3805).

Only 8.6% (329/3805) of preprints appeared in academic journals (as of May 2020), with a median of 28 days (interquartile range [IQR] 14–52; min–max: 0–117) from preprint posting to publication online. Observational studies (87.8%, n = 289/329) and RCTs (5.6%, n = 18/329) were most often published, while prevention and control (40.7%, n = 134/329) and diagnosis (31.6%, n = 104/329) were the most frequently mentioned research areas.

### 2. Characteristics of ongoing COVID-19-related research in the registers (trials and systematic reviews)

**Table 2** shows that 1621/339,863 (0.5%) records in Clinicaltrials.gov were COVID-19-related and that 652/32553 (2%) records in the Chinese Clinical Trial Registry (ChiCTR) were COVID-19-related. No other register had over 200 records of COVID-19-related research.

**Table 2:** Primary registries.

	**Publications**	
	COVID-19-related records No. (%)	Total no. of records
Clinicaltrials.gov (USA)	1561(0.5)	339,863
Chinese Clinical Trial Registry (ChiCTR)	648 (2.0)	32,553
EU Clinical Trials Register (EU-CTR)	196 (0.5)	37,185
Iranian Registry of Clinical Trials (IRCT)	195 (0.8)	24,573
Clinical Trials Registry—India (CTRI)	99 (7.0)	1475
German Clinical Trials Register (DRKS)	67 (1.7)	3959
Japan Primary Registries Network (JPRN)	64 (0.2)	28,794
Australian New Zealand Clinical Trials Registry (ANZCTR)	52 (0.2)	27,187
The Netherlands National Trial Register (NTR)	52 (0.6)	8613
International Standard Randomized Control Number (ISRCTN)	35 (0.4)	11,381
Brazilian Clinical Trials Registry (ReBec)	10 (0.2)	4085
Pan African Clinical Trial Registry (PACTR)	6 (0.5)	1162
Clinical Research Information Service (CRiS), Republic of Korea	2 (0.4)	505
Lebanese Clinical Trials Registry (LBCTR)	2 (3.0)	71
Peruvian Clinical Trial Registry (REPEC)	0 (0.0)	1849
Sri Lanka Clinical Trials Registry (SLCTR)	1 (0.3)	349
Thai Clinical Trials Registry (TCTR)	9 (3.0)	336
Cuban Public Registry of Clinical Trials(RPCEC)	19 (NA)	NA

**Table 3** presents the characteristics of the two primary registries with the highest number of COVID-19-related research studies. The most frequent study types were interventional studies in Clinicaltrials.gov and in ChiCTR (60% and 50%, respectively), with phase 3 the most frequent in Clinicaltrials.gov (15%). The most frequent recruiting status was “ongoing” in all registers (min-max: 47%-99%).

**Table 3:** Characteristics of primary registries with highest number of COVID-19-related trials. (absolute frequencies).

Characteristic		Clinicaltrials.gov N = 1621 no. (%)	Chinese Clinical Trial Registry (ChiCTR) N = 652 no. (%)	EU Clinical Trials Register N = 196 no. (%)
Country	Africa	56 (3.5)	-	-
	Central America	3 (0.2)	-	-
	East Asia (China)	120 (7.4)	652 (100.0)	-
	*Japan*	4 (0.2)	-	-
	Europe	628 (38.7)	-	196 (100.0)
	Middle east	83 (5.1)	-	-
	North America	380 (23.4)	-	-
	*Canada*	44 (2.7)	-	-
	*United States*	326 (20.1)	-	-
	*Mexico*	18 (1.1)	-	-
	North Asia	13 (0.8)	-	-
	Pacifica	10 (0.6)	-	-
	South America	48 (2.9)	-	-
	South Asia	20 (1.2)	-	-
	South-East Asia	18 (1.1)		-
**Study Type**	Interventional	940 (57.9)	323 (49.5)	-
	Observational	663 (40.9)	260 (39.9)	-
	Other (i.e., patient registries)	125 (7.7)	69 (10.6)	-
**Phase**	Early Phase 1	19 (1.2)	218* (33.4)	-
	Phase 1	91 (5.6)	13 (2.0)	6 (3.1)
	Phase 2	375 (23.1)	8** (1.2)	99 (50.5)
	Phase 3	241 (14.9)	3***(0.5)	75 (38.3)
	Phase 4	55 (3.4)	67 (10.3)	33 (16.8)
	Not applicable	282 (17.4)	212 (32.5)	-
**Recruiting status**	Recruiting	795 (49.0)	308 (47.2)	194 (98.9)
	Complete	76 (4.7)	53 (8.1)	0
	Suspended/temporarily halted	7 (0.4)	16 (2.5)	0
	Other (e.g. withdrawn)	8 (0.5)	275 (42.2)	1 (0.5)

Na*: records not available because not possible to retrieve.

Data were collected as reported in primary registers.

* phase 0 for ChiCTR.

**3 trials were phase I-II.

***1 trial was phase II-III.

**Table 4** presents the characteristics of the records retrieved from the PROSPERO database for COVID-19-related systematic reviews: 962 systematic reviews were registered; almost all focused on humans (99.7%); the two most frequent research areas were treatment (19.1%) and health impact (16.6%).

**Table 4:** Characteristics of COVID-19-related systematic review protocols.

**Characteristic**		**Prospero registration N = 962**	
		No.	%
**Species**	*Humans*	959	99.0
	*Animals*	3	0.3
**Research Area/Tag as reported in PROSPERO**	Chinese medicine	70	7.0
	Diagnosis	52	5.0
	Epidemiological	155	16.0
	Genetics	7	0.7
	Health impacts	160	17.0
	Mental health	76	8.0
	Other	31	3.0
	Personnel protective equipment	17	2.0
	Prognosis	50	5.0
	Public health	10	1.0
	Transmission	26	3.0
	Treatments	184	19.0
	Vaccines	3	0.3

## Discussion

During the first half of 2020 (about 5 months/21 weeks), the spread of COVID-19 matched the rapid and statistically significant increase in the number of publications appearing in academic journals and on preprint servers (3805 and 3635 records, respectively; *p*< 0.001). Clinicians, researchers, public health authorities, and other stakeholders (e.g., government administrators) need to be able to identify in a timely manner accurate and reliable health information from trustworthy data sources. With this study we analyzed the frequency and type of publication covering the initial scientific response to the COVID-19 pandemic as of 20 May 2020. In general, we noted that to fill the void left by the absence of primary studies, academic journals ran opinion pieces (i.e., letters, editorials, commentary), whereas preprint servers listed empirical studies, including epidemiological findings and research results in biology, infection diagnosis and prevention.

Our findings reflect the progressive advances in attempts to answer questions from the scientific community. While the total number of publications rose dramatically in the first months of the study period (not counting the last few weeks because of indexation delays), academic sources provided scarce higher-hierarchy evidence in the form of systematic reviews/meta-analysis (n = 29) or randomized controlled trials (n = 8). This observation is shared by previous studies that found few high-quality studies on COVID-19 [17, 29, 30]. Since there has been insufficient time to design, approve, conduct, and conclude such studies, reports of preliminary results can be expected over the upcoming months. A previous cross-sectional study on COVID-19-related publications revealed that only a quarter of the first 2118 articles contained original data [31]. In our study, the number of registered trial protocols listed as “ongoing” in Clinicaltrials.gov and in ChiCTR (n = 795 and n = 308, respectively) signals an upward trend. We retrieved a total of 2273 trials from the two registers and noted a phenomenal growth rate in both. As of 22 February 2020, merely 171 COVID-19-related interventional trials were registered (138 in ChiCTR and 33 in ClinicalTrials.gov) [32]; one month later (24 March 2020) the number was 614 COVID-19-related interventional trials (471 in ChiCTR and 143 in ClinicalTrials.gov) [33]. The rise may be linked to the breakthrough news from China and other countries that announced a successful drug treatment [34]. In retrospect, however, the announcement by Chinese scientists that made claims for chloroquine was not a true breakthrough in COVID-19 treatment; nonetheless, it could have attracted low-quality research and clinical practice [17, 35, 36].

Many of the systematic review protocols registered in PROSPERO (n = 962) are focused on treatment; however, they may encounter limitations due to the scarcity of the number or of the quality of clinical trials [17]. The secondary literature can be supported by indirect evidence from primary studies involving patients different from those of interest (i.e., COVID-19), as suggested by the Grading of Recommendations Assessment, Development and Evaluation (GRADE) approach [37]. The search for answers, treatments, and vaccines during a pandemic ought to be based on the highest levels of evidence such as systematic reviews and randomized controlled trials. Furthermore, companies and universities have begun to accelerate the discovery of experimental drugs and vaccines for COVID-19 through their research and development pipeline [38]. The World Health Organization (WHO)’s Blueprint list recognizes coronaviruses as infectious agents of priority importance: given the public health emergencies of international concern and the current absence of efficacious drugs and vaccines, research and development need to be accelerated if diseases caused by coronaviruses are to be controlled [39].

For the duration of the COVID-19 pandemic, several scientific journals allow access to publications through preprints posted online [40–42]. However, the credibility of preprints not published in a reputable peer-reviewed journal is uncertain. We found that only 8% of preprints were published in academic journals after undergoing peer review. We wonder whether time constraints on converting preprints into a final peer-reviewed version and then waiting for publication are the reasons for such a low percentage. The time to publication of a definitive manuscript can take up to 166 days for a preprint to be published with its DOI in a journal article [43]. One fourth of the published preprints we retrieved took between 52 and 117 days to appear in an academic journal. Publication setbacks, often due to bottlenecks in the peer-review process, can delay the timely dissemination of information in a global health emergency like the present COVID-19 pandemic [43]. Furthermore, unpublished studies generate a waste of effort and money invested in health and medical research globally [44, 45]. We noted that findings in areas like mathematics were published only on preprint servers like ArXiv [46, 47]. The potential harm from posting erroneous provisional research is one reason why the medical community was so cautious about preprints in the first place [48]. But it is interesting to observe that preprints are now gaining more attention and citations than the corresponding peer-reviewed article (i.e., academic publication) [49]. While it is still soon to analyze citation trends for COVID-19-related articles, the publication trends we observed (as of 20 May 2020) were remarkable for the greater number of preprints than academic publications and the higher percentage of empirical research reported in preprints. This observation contrasts with the findings by Lv et al. (as of 6 February 2020) who reported that academic journal articles accounted for 77.1% of study publications, preprints for 14.1%, while 8% were published only online [50]. A plausible explanation for these discrepancies is the sheer amount of recent empirical studies more rapidly accessible in preprint databases than in academic journals.

Preprints provide a faster and iterative alternative or complement to journal publication and the peer-review system. In addition, they catalyze biomedical discovery, support career advancement, and improve scientific communication [43]. If all scientific publishers endorsed preprint posting for research, it would serve as a preliminary step to publication and a clear signal to all scientists that preprints are an integral part of scientific communication [21]. In essence, better utilization of preprints would mean better fruition of trusted science. By tracking the preprint along the publication process the reliability of research can be controlled and differentiated from the final peer-reviewed publication. Also, it might also benefit the author and the scientific community alike: the former can share their research and attract immediate attention, while the latter can directly access the research paper, give feedback to improve the manuscript, and open a discussion leading to new ideas, follow-up studies or collaboration with other research groups [51]. This may be viewed as a necessary step toward a more open and transparent peer-review process, which forms the cornerstone of scientific activities and part of a system that ensures that only quality research papers are released into the scientific community [23]. Commentaries, letters to the editor, and other forms of non-research publications were largely found in academic journals. Though less suitable as preprints, they can still benefit from external peer-review since they are biased or present personal perspectives. Informed readers of empirical research can more objectively appraise the research methods, the data presented, the validity of its conclusions or limitations in either preprint or definitive versions.

The challenge in this emergency is to increase the amount of completed and published research and translate it into practice. However, a recent commentary in *International Immunopharmacology* stated, “quantity does not equal quality” [52]. While the dissemination of data is an essential part of the response, the detection of poor methodology and the retraction of clinical trials published in reputable journals are unfortunate examples of misconduct and rushed research in this pandemic [17, 53].

### Limitations of the study

This study covers only journals and publications indexed in PubMed, a comprehensive but not exhaustive database for biomedical literature. We searched only a few of the repositories and servers for posting preprints (i.e., bioRxiv and medRxiv). We used the COVID-19 section of these servers to investigate the preprint versions at the time of our data extraction. We collected data within a tightly defined window (December 2019 through May 2020). Since no filter nor topics exist for selecting preprint categories and the average time the servers take to display search results can be long [54], we performed hand screenings. This partly reduces the validity of the comparison of study types (academic vs. preprint). An illustrative example is that of the epidemiologic studies: we retrieved different study types in preprints (e.g., case-control studies, cross-sectional studies, biological and mathematical models), whereas when searching PubMed we used MeSH terms such as clustered study design (e.g., case-control studies, cross-sectional studies). In addition, epidemiological studies are not indexed in PubMed as a “publication type” like RCTs, for instance. This can affect the reliability, accuracy, and speed of indexation of epidemiological studies in PubMed. compared to preprint servers. It may also explain why many studies retrieved from PubMed were not yet classified by study type (the total sum of the articles indexed by study type was clearly below the total number of papers retrieved). Therefore, clear-cut publication types like RCTs or letters to the editors may have had faster, more reliable indexation. It is reasonable to infer that many of the studies without indexation for a publication or study type could be some format other than an RCT or commentaries/letters (e.g., epidemiologic, secondary data analysis, etc.). Although indexation of COVID-19-related research has been prioritized, delays may apply and these may have been visible in the data we obtained during the weeks of May 2020.

When needed (when the search filters or tags could not be reliably applied), one researcher performed manual coding and the other verified it rather than involving two independent researchers. This option is typical of rapid review types and a compromise to expedite the results for which timeliness is key, perhaps at the expense of a certain degree of uncertainty about the accuracy of the results. We did not adjust the characteristics for a country’s population size, for example using the World Bank’s data to facilitate comparison of the number of studies per country (https://data.worldbank.org/).

## Conclusion

The number of COVID-19-related publications has increased dramatically since the outbreak began in late 2019. The bulk of the scientific literature in the first five months of the epidemic comprised short reports of data, opinions or perspectives (e.g., letters to the editor or commentaries) or reported (secondary) data rather than primary data. Numerous clinical trials and systematic reviews are registered but their results have not yet been published. The focus of research is effective preventive/control and therapeutic strategies, reflecting the initial response by the research community. Preprints can provide readers with direct, rapid access to research findings. Despite the deluge of papers [55], screening and reviewing preprints might benefit both the authors and the scientific community, while informing the general public with timely access to scientific content and discussions that could boost scientific credibility [41].

In brief, we noted a huge increase in the amount of scientific papers within a short period of time: COVID-19 has aroused an unprecedented interest in research, a demand for answers, and rapid publication of findings. Nevertheless, efforts and time should be devoted to scientifically sound information rather than rushing to publish results that may not be fit to print. Overproduction of research is another form of waste that undermines public trust in science [56].

## Supporting information

S1 Appendix (DOCX)

## Abbreviation

COVID-19: Coronavirus disease 19
SARS-CoV-2: Severe Acute Respiratory Syndrome coronavirus 2
PROSPERO: international prospective register of systematic reviews
MeSH: Medical Subject Headings
RCTs: randomized controlled trials
