# Supplementary material for: Characteristics of academic publications, preprints, and registered clinical trials on the COVID-19 pandemic
Source: PLoS One. 2020 Oct 6;15(10):e0240123. doi: 10.1371/journal.pone.0240123 (PMC7537872; doi:10.1371/journal.pone.0240123)
Supplement: S1 Appendix — (DOCX) [file pone.0240123.s001.docx]

**S1. Appendix**

**Contents:**

[**Pubmed database** 1](#_Toc51751059)

[**Preprints MedRxiv and BioRxiv database** 4](#_Toc51751060)

[**Primary Registers** 5](#_Toc51751061)

[**PROSPERO** **Register** 5](#_Toc51751062)

## **Pubmed database**

This appendix details the search terms used in the PubMed searches as organized by sets of related content. Each set was appropriately combined (with the operator “AND”) with one or more of the other sets for each search conducted in PubMed. For example, for finding “COVID-19 research designs”, we combined the common filter (i.e. the one applied transversally to all searches), that for COVID-19, and finally that for systematic review. The same reasoning applied to all the searches.

Introductory technical notes:

- The organization of the MeSH tree and the detailed definitions and elements included within each MeSH term can be found and searched online at: <http://www.ncbi.nlm.nih.gov/mesh/1000048>. Lastly accessed: May 20th, 2020.
- Note we do not use the option to exclude the explosion of MeSH terms. Therefore, all MeSH terms that, within the MeSH tree, are under those indeed selected are searched for as well (e.g. “randomized controlled trials” contain “Equivalence Trial”) .
- In each search, we considered all publications indexed in PubMed up to May 20th, 2020.
- COVID-19 is an emerging, rapidly evolving situation. In March 2020, five new Medical Subject Heading (MeSH) Supplementary Concept Records (SCR Class 2-Protocol) were added to the 2020 MeSH Browser in response to the COVID-19 pandemic: COVID-19 diagnostic testing; COVID-19 drug treatment; COVID-19 serotherapy; COVID-19 vaccine; LAMP assay; These SCRs first appeared in the MeSH export files available on March 26, 2020 and March 28, 2020. Prior to March 2020, the SCR Class 2-Protocol type was limited to cancer protocols. To expedite the creation of MeSH vocabulary available for COVID-19, MeSH has expanded the use of the SCR Class 2-Protocol types to include protocols, treatments, and diagnostic methods for diseases. Each of these new SCRs (Class 2-Protocol) is assigned headings mapped to MeSH descriptors. See the "Heading Mapped to" field in the MeSH Browser record for the individual SCRs to identify the descriptors that are mapped to the SCRs. Three other COVID-19 related SCRs were added to the MeSH Browser prior to March 26, 2020: COVID-19; severe acute respiratory syndrome coronavirus 2; spike glycoprotein, COVID-19 virus. <https://www.nlm.nih.gov/pubs/techbull/ma20/brief/ma20_mesh_scr_novel_coronavirus_disease.html>

***SETs of Terms, by category***

**Common Set – used into all the searches, inclusively when searching the full PubMed database**

("COVID-19 vaccine" [Supplementary Concept] OR "spike protein, SARS-CoV-2" [Supplementary Concept] OR "COVID-19 diagnostic testing" [Supplementary Concept] OR "pediatric multisystem inflammatory disease, COVID-19 related" [Supplementary Concept] OR "COVID-19 serotherapy" [Supplementary Concept] OR "nucleocapsid protein, Coronavirus" [Supplementary Concept] OR "COVID-19" [Supplementary Concept] OR "severe acute respiratory syndrome coronavirus 2" [Supplementary Concept] OR "membrane protein, SARS-CoV-2" [Supplementary Concept] OR "envelope protein, SARS-CoV-2" [Supplementary Concept] OR "ORF1ab polyprotein, SARS-CoV-2" [Supplementary Concept] OR "3C-like proteinase, Coronavirus" [Supplementary Concept] OR "ORF8 protein, SARS-CoV-2" [Supplementary Concept] OR "ORF7a protein, SARS-CoV-2" [Supplementary Concept] OR "ORF6 protein, SARS-CoV-2" [Supplementary Concept] OR "ORF7b protein, SARS-CoV-2" [Supplementary Concept] OR "ORF3a protein, SARS-CoV-2" [Supplementary Concept] OR "RNA-dependent RNA polymerase, coronavirus" [Supplementary Concept] OR "NSP9 protein, SARS-CoV-2" [Supplementary Concept] OR "LAMP assay" [Supplementary Concept] OR "COVID-19 drug treatment" [Supplementary Concept] OR "nidoviral uridylate-specific endoribonuclease" [Supplementary Concept] OR "NSP1 protein, SARS-CoV-2" [Supplementary Concept] OR "NSP3 protein, SARS-CoV-2" [Supplementary Concept] OR "NSP5B protein, SARS-Cov-2" [Supplementary Concept] OR "NSP5A protein, SARS-Cov-2" [Supplementary Concept] OR "NSP4 protein, SARS-CoV-2" [Supplementary Concept] OR "NSP10 protein, SARS-CoV-2" [Supplementary Concept] OR "NSP6 protein, SARS-CoV-2" [Supplementary Concept] OR "NSP7 protein, SARS-CoV-2" [Supplementary Concept] OR "NSP8 protein, SARS-CoV-2" [Supplementary Concept] OR "nsp2 protein, SARS-CoV-2" [Supplementary Concept] OR "papain-like protease, Coronavirus" [Supplementary Concept]) **AND** (y_1[Filter]) Filters: in the last 1 year

***Research species for:***

- Other Animal: NOT “Humans”[Mesh]
- “Humans”[Mesh]

***Research designs individually searched for:***

- **Letter**

"Letter" [Publication Type]

- **Case reports**

"Case Reports"[ptyp]

- **Epidemiologic Studies**

"Epidemiologic Studies"[Mesh]

- **Randomized Controlled Trials**

"Randomized Controlled Trial" [ptyp]

- **Systematic Reviews**

((Review[ptyp] AND systematic[tw] AND systematic[sb]) OR "Cochrane Database Syst Rev"[Journal] OR (“systematic review”[ti] OR “scoping review”[ti] OR “realist review”[ti])))

***Research area individually searched for:***

- **Vaccine**

"Vaccines"[Mesh]

- **Drug treatment – management of COVID-19**

"Drug Therapy"[Mesh]

- **Diagnosis**

"Diagnosis"[Mesh]

- **Prevention and control (e.g., Masks, social distancing)**

"Infection Control"[Mesh] OR "Pandemics/prevention and control"[Mesh] OR "Coronavirus Infections/prevention and control"[Mesh]

- **Rehabilitation (e.g., Pulmonary rehabilitation)**

"Rehabilitation"[Mesh]

- **Prognosis**

“Prognosis[Mesh]”

- **Biology/Genetics**

"Biology"[Mesh]

## **Preprints MedRxiv and BioRxiv database**

We selected the records in the included the dedicated section about COVID-19. We searched for COVID-19 related preprints, defined as preliminary reports of work that have not been certified by peer review, in MedRxiv and bioRxiv database (<https://connect.biorxiv.org/relate/content/181>). We selected these two database since are the most popular on coronavirus research by Nature on 14 May 2020 (1) and are endorsed by several indexed journals.

For selecting studies we took advantages from the following link <https://observablehq.com/@ismms-himc/covid-19-sars-cov-2-preprints-from-medrxiv-and-biorxiv> to A PrIISM scientist, a dedicated website hosting all the updated citations for a shared pre-publication reviewing initiative. Then manuscripts are examined by volunteer academics or subject specialists who scan for non-scientific content and health or biosecurity risks. BioRxiv mainly uses principal investigators; medRxiv uses health professionals. On bioRxiv, this is usually completed within 48 hours. On medRxiv, papers are scrutinized more closely because they may be more directly relevant to human health, so the turnaround time is typically four to five days (<http://www.wame.org>).

## **Primary Registers**

We searched all primary registers for ongoing studies on COVID-19. Worldwide, there is growing number of registries. Primary Registries in the WHO Registry Network meet [specific criteria](https://www.who.int/ictrp/network/criteria_summary/en/) for content, quality and validity, accessibility, unique identification, technical capacity and administration. We investigated all Primary Registries meet the requirements of the [ICMJE](http://www.icmje.org/) according to the WHO Registry Network (<https://www.who.int/ictrp/network/primary/en/>):

We selected the records in the section dedicated about “COVID-19, coronavirus, SARS-COV-2”. About the first three registers with the most volume of research were investigated specific characteristics using the Filters offered by each register: country, study type, phase, and recruitment status.

## **PROSPERO** **Register**

We follow the search strategy proposed by the dedicated section into PROSPERO database and here reported:

(((coronavirus or corona-virus) AND (wuhan or beijing or shanghai or Italy or South-Korea or korea or China or Chinese or 2019-nCoV or nCoV or COVID-19 or Covid19 or SARS-CoV* or SARSCov2 or ncov)) OR (pneumonia AND Wuhan) or "COVID-19" or "2019-nCoV" or "SARS-CoV" or SARSCOV2 or 2019-nCov or "2019 coronavirus" or "2019 corona virus" or covid19 or ncov OR "novel corona virus" or "new corona virus" or "nouveau corona virus" or "2019 corona virus" OR "novel coronavirus" or "new coronavirus" or "nouveau coronavirus" or "2019 coronavirus").

For specific characteristics we used the Filters offered by the PROSPERO database: Animals, Humans, Chinese medicine, Diagnosis, Epidemiological, Genetics, Health impacts, Mental health, Other, PPE, Prognosis, Public health, Transmission, Treatments, Vaccines.

**References**

1. Diana K. How preprint servers are blocking bad coronavirus research. Nature. 2020;581:130-1
